# Single cells and TRUST4 reveal immunological features of the HFRS transcriptome

**DOI:** 10.3389/fmed.2024.1403335

**Published:** 2024-05-13

**Authors:** Ran Xiao, Mu Lin, Mubo Liu, Qingqing Ma

**Affiliations:** The Central Laboratory of Guizhou Aerospace Hospital, Zunyi, China

**Keywords:** hemorrhagic fever with renal syndrome, T cell receptor, B cell receptor, scRNA-seq, hdWGCNA

## Abstract

The etiology of hemorrhagic fever with renal syndrome (HFRS) is significantly impacted by a variety of immune cells. Nevertheless, the existing techniques for sequencing peripheral blood T cell receptor (TCR) or B cell receptor (BCR) libraries in HFRS are constrained by both limitations and high costs. In this investigation, we utilized the computational tool TRUST4 to generate TCR and BCR libraries utilizing comprehensive RNA-seq data from peripheral blood specimens of HFRS patients. This facilitated the examination of clonality and diversity within immune libraries linked to the condition. Despite previous research on immune cell function, the underlying mechanisms remain intricate, and differential gene expression across immune cell types and cell-to-cell interactions within immune cell clusters have not been thoroughly explored. To address this gap, we performed clustering analysis on 11 cell subsets derived from raw single-cell RNA-seq data, elucidating characteristic changes in cell subset proportions under disease conditions. Additionally, we utilized CellChat, a tool for cell–cell communication analysis, to investigate the impact of MIF family, CD70 family, and GALECTIN family cytokines—known to be involved in cell communication—on immune cell subsets. Furthermore, hdWGCNA analysis identified core genes implicated in HFRS pathogenesis within T cells and B cells. Trajectory analysis revealed that most cell subsets were in a developmental stage, with high expression of transcription factors such as NFKB and JUN in Effector CD8+ T cells, as well as in Naive CD4+ T cells and Naive B cells. Our findings provide a comprehensive understanding of the dynamic changes in immune cells during HFRS pathogenesis, identifying specific V genes and J genes in TCR and BCR that contribute to advancing our knowledge of HFRS. These insights offer potential implications for the diagnosis and treatment of this autoimmune disease.

## 1 Introduction

Hemorrhagic fever with renal syndrome epitomizes a zoonotic malady characterized by acute renal insufficiency, hypotension, and coagulopathy (1). The causative agents behind HFRS encompass Hantaan virus (HTNV), Dobrava virus (DOBV), Seoul virus (SEOV), Puumala virus (PUUV), and Amur virus (AMV), each manifesting varying degrees of pathogenicity. China stands as an endemic epicenter, with HFRS instances linked to HTNV and SEOV constituting a staggering 90% of the global caseload (2). Analogous to other viral afflictions, the pathogenesis of HFRS predominantly hinges on orchestrated immune responses, encompassing both innate and adaptive arms, aimed at eliminating the infective agent. Consequently, immune-mediated pathways, such as immune complex formation, complement activation, T cell activation, B cell responses, and HTNV-induced cytokine cascades, are widely acknowledged as pivotal contributors to HFRS pathophysiology (3). Studies have unveiled a correlation between the levels of HTNV-specific CD8+ T lymphocytes and distinct phases of HFRS, notably observing a significant surge during the febrile phase (4). Moreover, unraveling the intricacies of T and B cell receptor diversity holds paramount importance in the realm of infectious disease research and therapeutics (5, 6). However, the precise diversity profiles of TCR and BCR in the context of HFRS remain elusive.

Currently, the computational tool TRUST4 is deployed to discern immune signatures from bulk RNA-seq data, facilitating the construction of TCR and BCR libraries pertinent to the disease (7). Given the accessibility of peripheral blood samples from patients, they serve as common clinical transcriptome sequencing samples to unveil alterations in disease onset and progression (8). Hence, the objective of this study is to comprehensively delineate the attributes of TCR and BCR in HFRS using peripheral blood samples from patients.

Single-cell RNA sequencing (scRNA-seq) furnishes a nuanced understanding of the transcriptomic diversity among individual cells, affording insights into the developmental trajectories of immune cells and their intricate gene expression profiles (9, 10). This technology allows for the elucidation of distinct gene expression signatures within immune cells at a granular level, shedding light on the dynamic interplay between these cells and offering invaluable perspectives for dissecting disease mechanisms and devising therapeutic interventions.

In this study, we harnessed the power of TRUST4 and scRNA-seq to elucidate the immunological intricacies of HFRS patients, unraveling gene modulations and intercellular dialogues among diverse immune cell subpopulations. These findings pave the way for innovative diagnostic and therapeutic strategies in the management of HFRS.

## 2 Materials and methods

### 2.1 Data collection

Bulk RNA-sequencing data: We obtained the gene expression profiles (RNA-seq) of blood from patients (GSE158712), encompassing HFRS samples (SRR12739155, SRR12739156, SRR12739157, SRR12739162, SRR12739163, SRR12739164) and healthy control (HC) samples (SRR12739152, SRR12739153, SRR12739154). PBMC scRNA-seq data(GSE161354) of two healthy samples and six HFRS patients were obtained from GSM4905210, GSM4905211, GSM4905212, GSM4905213, GSM4905214, GSM4905215, GSM4905216, GSM4905217. The clinical information of the samples is shown in Table 1.

**Table 1:** Basic information of single cell data samples.

Samples	Age	Gender	Disease state
GSM4905210	52	Male	HFRS fever stage
GSM4905211	34	Male	HFRS fever stage
GSM4905212	49	Male	HFRS fever stage
GSM4905213	29	Male	HFRS fever stage
GSM4905214	37	Male	HFRS fever stage
GSM4905215	26	Male	HFRS fever stage
GSM4905216	32	Female	Normal health control
GSM4905217	29	Male	Normal health control

### 2.2 T-cell receptor and B-cell receptor repertoire construction and analysis

To establish TCR and BCR repertoires, we utilized the highly efficient tool TRUST4, specifically designed for reconstructing immune receptor repertoires from bulk RNA-seq data in both T cells and B cells. Following the standardized TRUST4 pipelines, we obtained comprehensive output data encompassing conventional TCR and BCR repertoire information. Subsequently, we conducted statistical analysis in the following manner: (1) Calculation of the relative frequency of all clonotypes within the TCR and BCR repertoires. To assess differences between the HFRS and HC groups, we employed Student’s *t*-test. (2) TCR β-chain and BCR heavy-chain consist of variable and constant regions, where the variable regions hold responsibility for antigen recognition and binding specificity. The β-chain variable region comprises three gene segments, namely variable (V), diversity (D), and junctional (J). The V(D)J rearrangement event gene encodes the variable region. Thus, the combination of V and J genes reflects clonotype diversity in both T-cell receptors and B-cell receptors. Proportions of various TRBV, TRBJ, IGHV, and IGHJ genes were calculated under both HFRS and HC conditions. Subsequently, Student’s *t*-test was employed to identify significantly altered TRBV, TRBJ, IGHV, and IGHJ genes. (3) Evaluation of the diversity of TCR and BCR complementary determining region 3 (CDR3) amino acid sequences using Chao1 and InvSimpson indices, conducted for TCR β-chain and BCR heavy-chain CDR3 amino acid sequences under both HFRS and HC conditions. (4) Analysis of the distribution of TCR β-chain and BCR heavy-chain CDR3 amino acid sequence lengths within the HFRS and HC groups. (5) Examination of the top 10 TCR and BCR V region motifs within the HFRS group.

### 2.3 Cell filtering and data normalization

This study accounted for the gene count, unique molecular identifier count, and mitochondrial gene percentage of each cell sample in the dataset to mitigate the impact of dead cells and cellular debris. Cells exceeding a total gene count of 2,500, falling below a gene count of 200, and exhibiting a mitochondrial gene percentage below 5% were excluded. The normalization procedure employed the LogNormalize method, which logarithmically transforms and standardizes the gene expression values of each cell, thereby ensuring consistent total RNA expression across all cells (Supplementary Figure S1).

### 2.4 Data dimension reduction and UMAP clustering analysis

The data normalization process is conducted using the ScaleData function, while the RunPCA function diminishes the dimensionality of the normalized data through principal component analysis (PCA), resulting in the generation of PCA outcomes that are subsequently printed. The FindNeighbors function is employed to compute the intercellular neighbor relationships, evaluating the distances between each cell and others based on the selected principal fraction (pcSelect), thereby identifying the nearest neighbor for each cell. The FindClusters function applies the Leiden algorithm to partition the cells into distinct clusters. The Leiden algorithm, a graph-based clustering technique, utilizes the previously constructed adjacency graph to determine the clustering. In this study, the data were segregated into different clusters based on a resolution parameter of 2.0. Higher resolution parameters yield more finely divided clusters, while lower resolution parameters classify a greater number of cells into a single cluster. Lastly, the RunUMAP function calculates the UMAP coordinates for each cell, utilizing the chosen principal fraction (pcSelect) to map the high-dimensional data to a two-dimensional space.

### 2.5 Identification of marker genes

To identify genes with high variability, we employed variable gene selection on a per-sample basis. Subsequently, an integration analysis was conducted on each sample to identify the top 2,000 genes exhibiting the greatest mean and dispersion across all samples. This selection was made to enhance subsequent analysis steps, including clustering and differential analysis of the data.

### 2.6 Cell annotation

Cell annotation was carried out through a meticulous manual annotation process. Additionally, the “FindAllMarkers” function was employed to identify specific markers for each cluster. This approach facilitated the initial classification of clusters based on the distinctive gene expression patterns of particular cell types, allowing for the annotation of cells using multiple reference sets.

### 2.7 hdWGCNA analysis

The high-dimensional weighted gene co-expression network analysis (hdWGCNA) was utilized to construct a scale-free network at the single-cell level using the R package “hdWGCNA”. A threshold of ≥0.80 was set to ensure a good fit to the scale-free topology model, and a soft threshold of 8 was selected to achieve optimal connectivity. The modules were assigned to the scRNA cohort using Ucell. To generate a protein–protein interaction (PPI) network, the “HubGeneNetworkPlot” function was employed.

### 2.8 Cell-cell communication analysis

To investigate intercellular communication, we utilized CellChat (version 1.6.1), an R package designed to facilitate the exploration of ligand-receptor interactions at the cell surface, thereby elucidating cell signaling across different cell types (11). By leveraging gene expression data, we inferred protein expression and established a comprehensive cell interaction network. Initially, we extracted the expression matrix and cell classification information from the dataset. Subsequently, the “createCellChat” function was employed to generate a CellChat object, enabling the calculation of communication probabilities and the inference of cell interaction networks. To ensure the reliability of the communication relationships, a filtering step was implemented. Specifically, communication relationships involving low-quality cells were excluded, with a minimum threshold of 3 cells set to eliminate unreliable or spurious communication signals. Additionally, we explored cell communication at the level of signaling pathways, allowing for the inference of communication between cells based on the involvement of specific signaling pathways. By aggregating cells, we computed the communication network for this higher-level analysis, providing a broader understanding of the interactions between different cell types and their respective communication pathways.

### 2.9 Trajectory analysis of single cells

For trajectory analysis, we utilized the Monocle2 software package (version 2.28.0). The metadata from the integrated Seurat object and the top 2000 variable genes from the integrated assay were imported into Monocle2.

### 2.10 SCENIC analysis

To investigate the enrichment of key transcriptomic factors in macrophage clusters, we utilized pySCENIC (version 1.2.4). The SCENIC dataset motif Hg38 was selected, and a co-expressed gene model was constructed by randomly selecting cells. GENIE3 was employed to identify the potential target genes of transcription factors. Furthermore, DNA-motif enrichment analyses were conducted using RcisTargetn (version 1.14.0) to identify direct binding sites, also known as regulons. The activity of each regulon in every cell was assessed using AUCell (version 1.16.0), which involved calculating the area under the receiver operating characteristic curve (AUC) and integrating the expression rank of all genes within the regulon. The resulting RegulonAUC matrix was imported into Seurat for cluster analysis and visualization of the single-cell data. Through this comprehensive analytical pipeline, we were able to explore the enrichment patterns of key transcriptomic factors in macrophage clusters. The integration of diverse computational tools allowed us to identify potential target genes, assess regulon activity, and facilitate the clustering analysis and visualization of the single-cell data using Seurat.

### 2.11 Statistical analysis

The analysis was conducted using the R software. The Seurat package was employed for data preprocessing and analysis. The data was normalized using the LogNormalize function, followed by scaling using the ScaleData function. Principal Component Analysis (PCA) was performed using the RunPCA function to reduce the dimensionality of the data. The cells were then classified into distinct clusters using the FindClusters function based on the PCA results. UMAP coordinates for each cell were computed using the RunUMAP function. To identify markers within each cell cluster, the FindAllMarkers function was utilized, aiding in the annotation of cell types. Statistical significance was determined using the Wilcoxon rank-sum test, with a significance level of *p* < 0.05.

## 3 Results

### 3.1 T-cell receptor repertoire analysis

**Figure 1:**
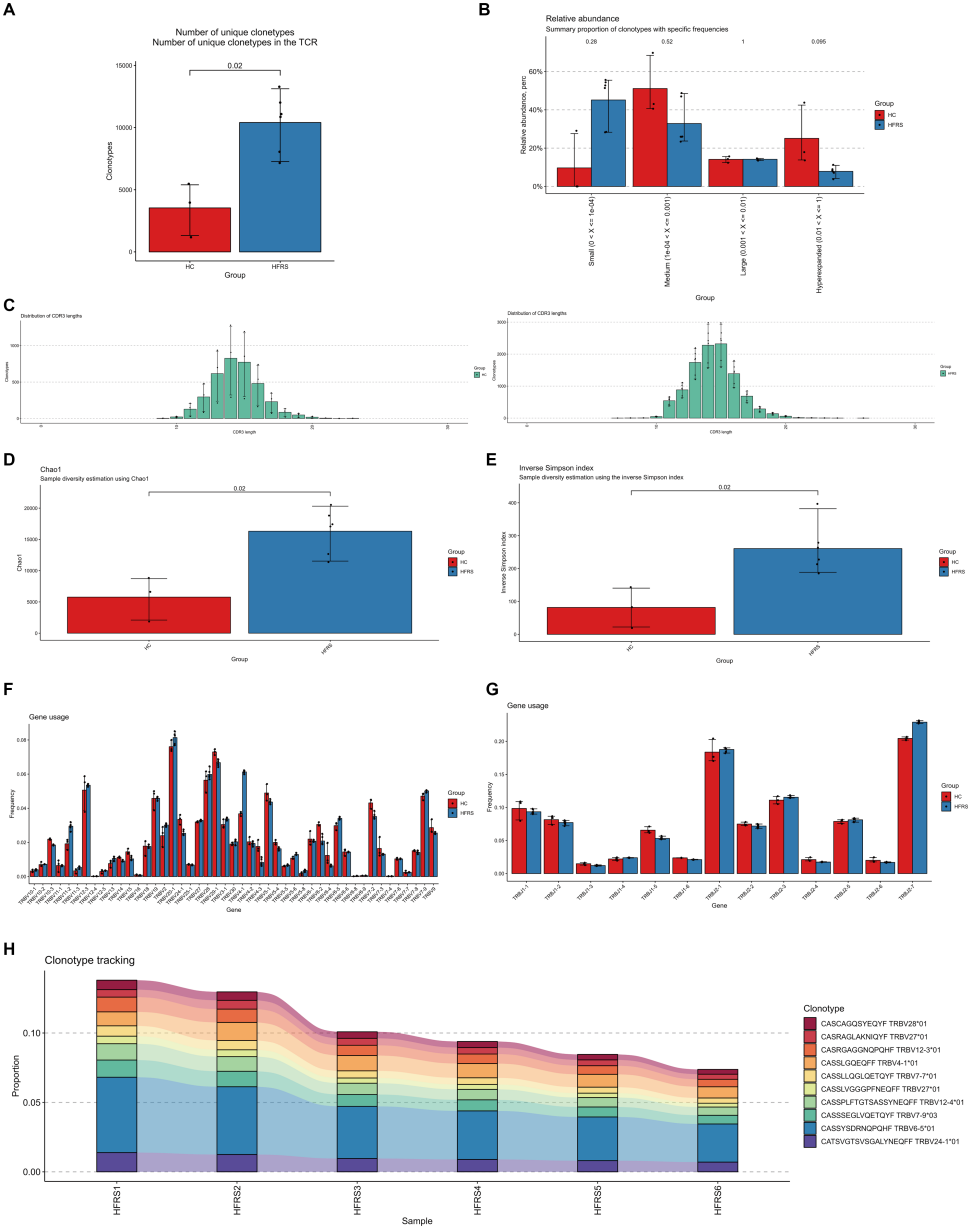
Characteristics of HFRS Immunological Repertoire (TCR Repertoire). **(A)** Comparison of clone count in the TCR repertoire. Clonotypes are defined based on the nucleotide sequence of the CDR3 region. Each CDR3 sequence defines a unique clonal population. **(B)** Comparison of clonal space equilibrium. Clonal space equilibrium analysis relative abundance, also known as clonal space equilibrium, is defined as the proportion of the library occupied by clonal populations with specific abundances. **(C)** Distribution of TCR CDR3 amino acid sequence lengths in HFRS and HC groups. **(D)** Differential comparison of clonotype diversity index Chao1. **(E)** Differential comparison of clonotype diversity index inverse Simpson index. **(F)** Comparison of TRBV gene usage. **(G)** Comparison of TRBJ gene usage. **(H)** Clonotype tracking in HFRS samples. Mean target values, error target values, standard errors, and *p*-values were obtained using Wilcoxon test.

Using the standard workflow of the TRUST4 algorithm, we extracted peripheral blood TCR libraries from both HFRS and HC groups based on extensive RNA-seq data. After obtaining the immunophenotype of T-cell receptors, we compared the clonotypes between the two groups. In comparison to the HC group, the clonotype frequencies were significantly increased in the HFRS group (*p* < 0.05, Figure 1A). Frequencies were categorized into small frequency (>0 and ≤ 0.0001), medium frequency (>0.0001 and ≤ 0.001), large frequency (>0.001 and ≤ 0.01), and super-expanded frequency (>0.01 and ≤ 1) for both groups. We observed an elevated distribution of small-frequency clonotypes and a reduced distribution of medium to super-expanded frequencies in the HFRS group (Figure 1B). These findings suggest that the HFRS group exhibits higher TCR repertoire diversity and greater clonal expansion, indicating a more specific T-cell response to HFRS. We also analyzed the distribution of the CDR3 amino acid sequence length in both groups. The most common CDR3 amino acid sequence lengths for HFRS and HC were 15 and 14, respectively. The difference in CDR3 amino acid sequence lengths between the two groups was not statistically significant (Figure 1C). Diversity assessments of CDR3 amino acid sequence were conducted using the Chao1 and inverse Simpson indices. Comparing the HFRS group to the HC group, both the Chao1 and inverse Simpson indices were significantly higher (*p* < 0.05, Figures 1D,E).

To assess the frequency of TRBV and TRBJ genes in both groups, the common usage frequencies of these genes were described in a bar chart (Figures 1F,G). Among the 27 different TRBV families, BV4-1 was significantly higher in the HFRS group compared to the HC group, while BV4-3, BV7-2, and BV6-2 were significantly lower in the HFRS group (*p* < 0.05). Similarly, TRBJ1-5 was decreased, and TRBJ20-1 was significantly increased in the HFRS group compared to the HC group (*p* < 0.05).

The V region of TCR is the most variable region in TCR molecules and plays a crucial role in determining the antigen-binding specificity of TCRs. We further examined the top 10 TCR V region germline sequences in the HFRS group, which were highly expressed in individuals. This result is attributed to the immune response triggered by HTNV virus antigen stimulation in the HFRS group (Figure 1H).

### 3.2 B-cell receptor repertoire analysis

**Figure 2:**
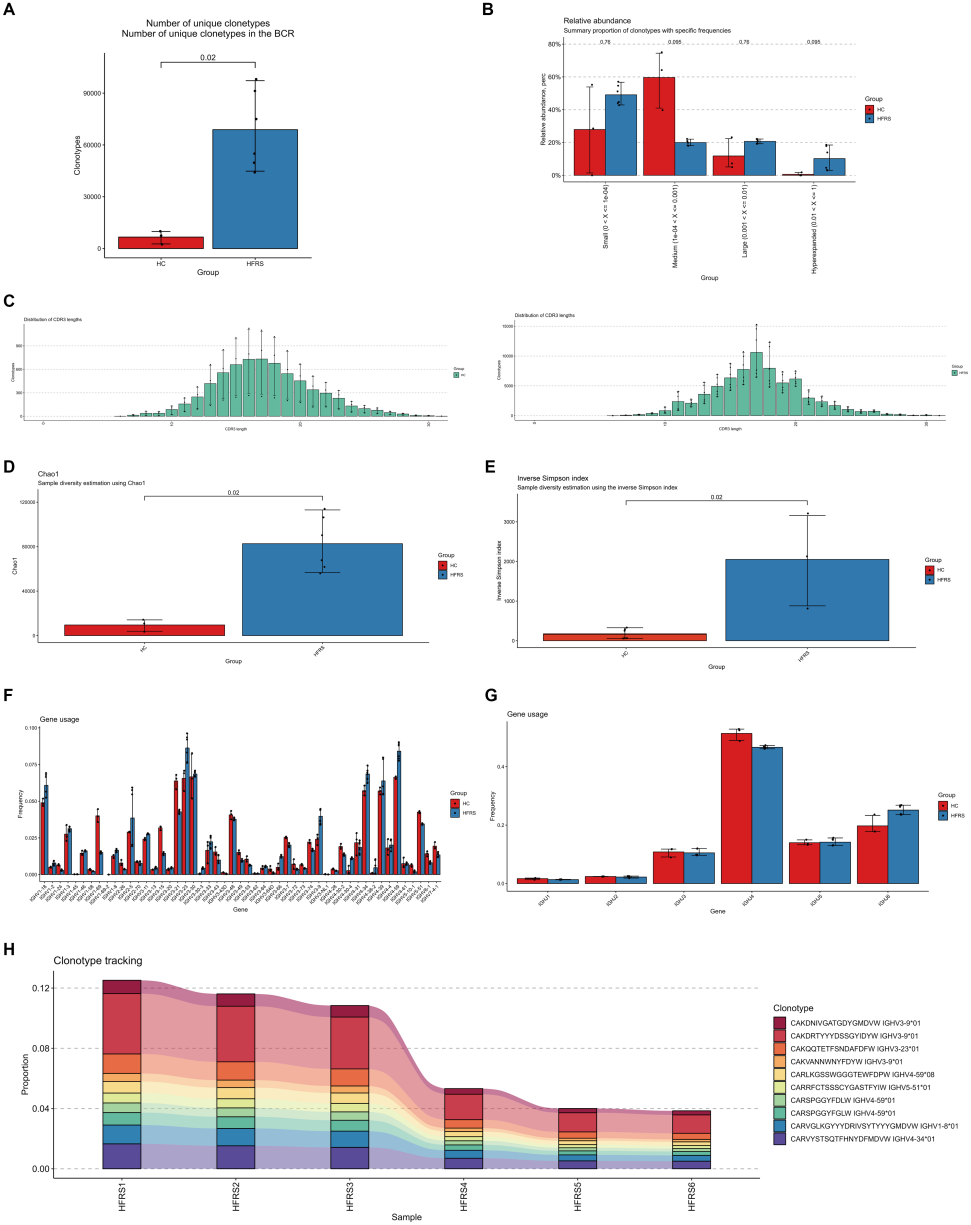
Characteristics of HFRS Immunological Repertoire (BCR Repertoire). **(A)** Comparison of clone count in the BCR repertoire. Clonotypes are defined based on the nucleotide sequence of the CDR3 region. Each CDR3 sequence defines a unique clonal population. **(B)** Comparison of clonal space equilibrium. Clonal space equilibrium analysis relative abundance, also known as clonal space equilibrium, is defined as the proportion of the library occupied by clonal populations with specific abundances. **(C)** Distribution of BCR CDR3 amino acid sequence lengths in HFRS and HC groups. **(D)** Differential comparison of clonotype diversity index Chao1. **(E)** Differential comparison of clonotype diversity index inverse Simpson index. **(F)** Comparison of IGHV gene usage. **(G)** Comparison of IGHJ gene usage. **(H)** Clonotype tracking in HFRS samples. Mean target values, error target values, standard errors, and *p*-values were obtained using the Wilcoxon test.

Similarly, using the standard workflow of the TRUST4 algorithm, we extracted peripheral blood BCR libraries from both the HFRS and HC groups based on extensive RNA-seq data. After obtaining the immunophenotype of B-cell receptors, we compared the clonotypes between the two groups. In comparison to the HC group, the clonotype frequencies were significantly increased in the HFRS group (*p* < 0.05, Figure 2A). Frequencies were categorized into small frequency (>0 and ≤ 0.0001), medium frequency (>0.0001 and ≤ 0.001), large frequency (>0.001 and ≤ 0.01), and super-expanded frequency (>0.01 and ≤ 1) for both groups. We observed an elevated distribution of small, large, and super-expanded frequency clonotypes, while the distribution of medium-frequency clonotypes was reduced in the HFRS group (Figure 2B). These results suggest that the HFRS group exhibits higher BCR repertoire diversity and greater clonal expansion, indicating a more specific B-cell response to HFRS. We also analyzed the distribution of the CDR3 amino acid sequence length in both groups. The most common CDR3 amino acid sequence lengths for HFRS and HC were 17 and 17, respectively. The difference in CDR3 amino acid sequence lengths between the two groups was not statistically significant (Figure 2C). Diversity assessments of CDR3 amino acid sequence were conducted using the Chao1 and inverse Simpson indices. Comparing the HFRS group to the HC group, both the Chao1 and inverse Simpson indices were significantly higher (*p* < 0.05, Figures 2D,E).

To assess the frequency of IGHV and IGHJ genes in both groups, the common usage frequencies of these genes were described in a bar chart (Figures 2F,G). Among the 27 different IGHV families, IGHV1-18, IGHV3-23, IGHV3-9, and IGHV4-59 were significantly higher in the HFRS group compared to the HC group, while IGHV1-69 and IGHV3-21 were significantly lower (*p* < 0.05). Similarly, in comparison to the HC group, IGHJ4 was decreased, and IGHJ6 was significantly increased in the HFRS group (*p* < 0.05).

The V region of BCR is the highly variable region in BCR molecules and plays a critical role in determining the antigen-binding specificity of BCRs. We further examined the top 10 BCR V region germline sequences in the HFRS group, which were significantly expressed in individuals. This result is attributed to the immune response triggered by HTNV virus antigen stimulation (Figure 2H).

### 3.3 Single-cell transcriptome of HFRS patient PBMCs

**Figure 3:**
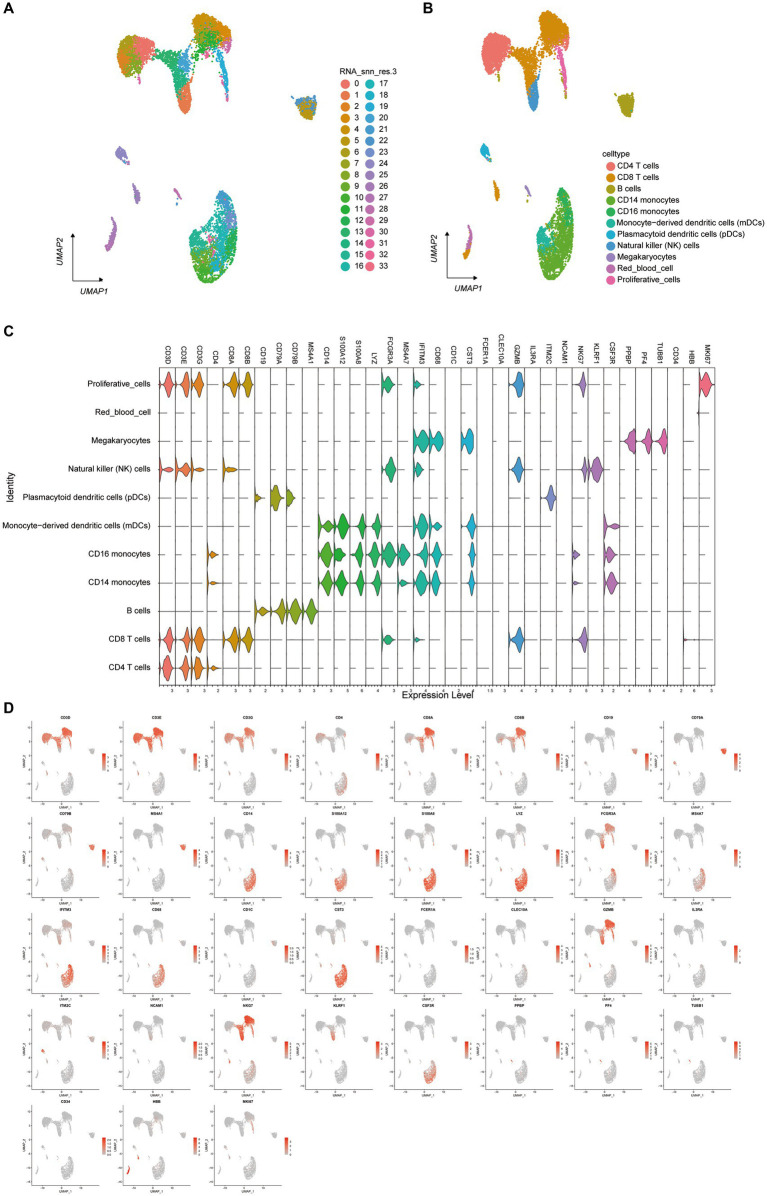
Single-Cell Transcriptome of PBMCs from HFRS and HC Groups. **(A)** Identification of cell clusters. Sequencing was performed on PBMC samples from the HFRS group (*n* = 6) and the HC group (*n* = 2). After quality control, 33 cell clusters were identified using UMAP. Each point corresponds to a single cell, colored according to cell type. Each color represents a distinct cluster. **(B)** UMAP plot of single cells. The 33 cell clusters were further identified as 11 cell types. UMAP was used to identify and visualize these 9 cell types. Each point represents an individual cell, colored based on its respective cell type. **(C)** Typical cell markers for 12 cell identity clusters, as shown in violin plots. **(D)** Typical cell markers were used to assign cell identities to the clusters represented in the UMAP plot. Data points are color-coded based on expression levels, with the legend marked on a logarithmic scale.

To investigate the single-cell transcriptome of PBMCs in HFRS patients, scRNA-seq data from 6 HFRS patients and 2 healthy individuals were analyzed. The individual cells from the 8 samples underwent integration, dimensional reduction, and clustering through an unsupervised method. Visualization via UMAP revealed 33 distinct cell clusters (Figure 3A). By assessing the expression of characteristic gene markers, we identified 11 clusters representing various cell types. These cell types encompassed CD4 (+) T cells (CD4+ CD3D+ CD3E+ CD3G+), CD14 (+) monocytes (CD14 + LYZ + S100A8 +), B cells (CD79A + MZB1 + CD38 + IGKC +), CD8 (+) T cells (CD3D+ CCR7 + LEF1 + CD8B+), red blood cells (HBD+ CA1 + HBA1 +), proliferative cells (STMN1+ MK167+), PDCs (IGHV7-4-1+), NK cells (KLRF1+ KLRB1 + MYOM2 +), mDCs (S100A8+ S100A12 +), megakaryocytes (PF4 + PPBP +), and CD16 monocytes (C1QA+ C1QB) (Figure 3B). All characteristic marker genes are depicted in Figure 3D, while the expression levels of annotated marker genes are presented in the violin plots (Figure 3C).

### 3.4 Phenotypic features of cell types in HFRS and HC groups

**Figure 4:**
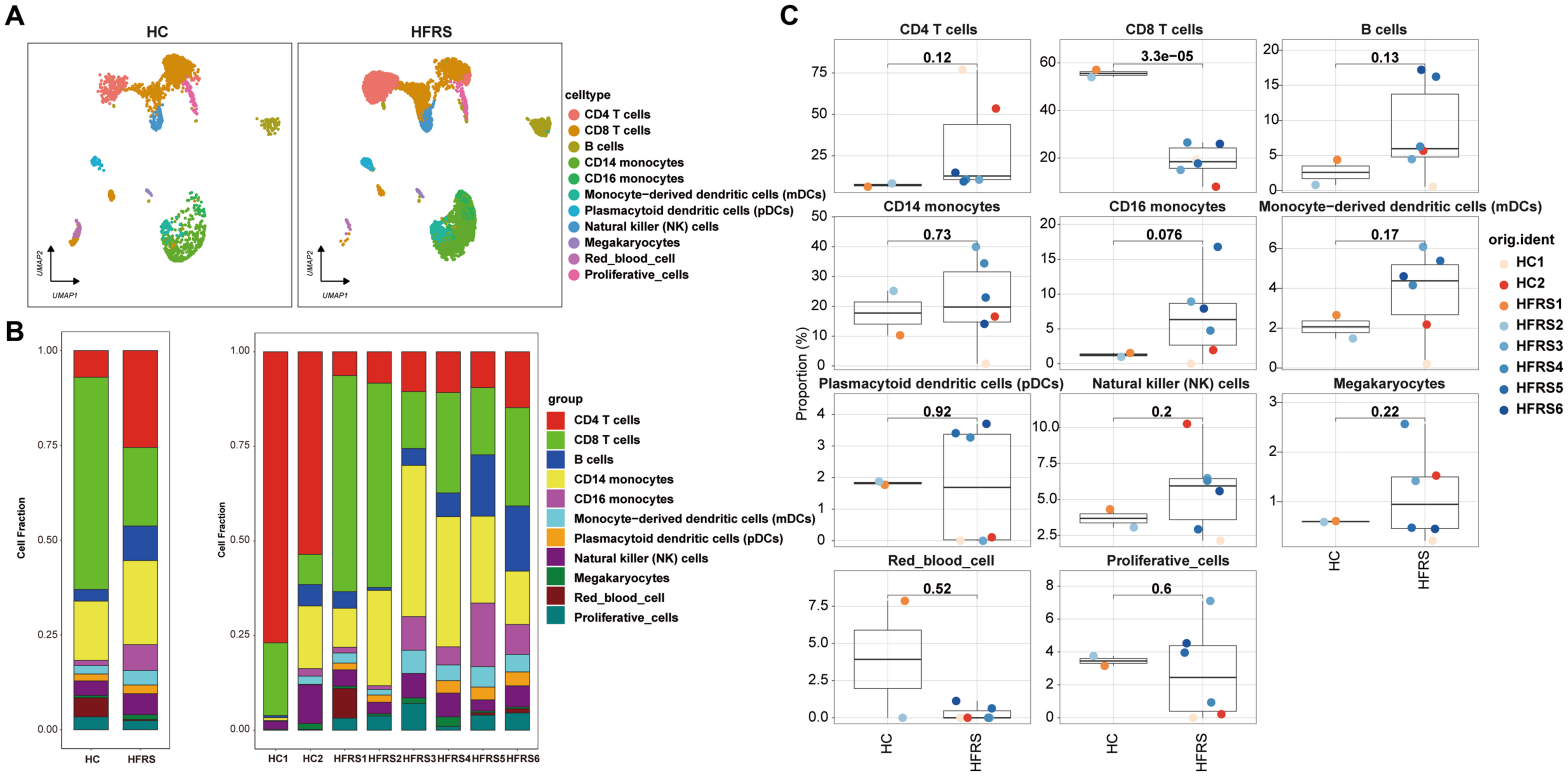
Differences in Cell Composition between HC and HFRS Groups. **(A)** UMAP projection of the HC and HFRS groups. Each point represents a cell, colored according to cell type. **(B)** The average proportion of each cell type in the HC and HFRS groups is provided. The bar plot on the left illustrates the average proportion of each cell type in both groups, calculated as (number of specific cell clusters in one group) / (total number of cells in one group). The plot on the right displays the proportion of each PBMCs cell subset in each sample, calculated as (number of specific cell clusters in one sample) / (total number of cells in one sample). **(C)** Box plots showing the distribution of cell composition in the two groups (*n* = 2 in the HC group, *n* = 6 in the HFRS group).

To scrutinize the disparities in cellular composition between the HFRS and HC cohorts, Figure 4A delineates the expression profiles of the 11 distinct cell types in each group. Employing scRNA-seq data, we computed the relative proportions of each cell type within individuals from both cohorts (Figures 4B,C). Notably, juxtaposed with the HC cohort, the HFRS group exhibited a conspicuous diminution in the relative proportions of CD8 T cells, CD4 T cells, and CD16 monocytes, while the relative proportions of other cell types remained relatively unaltered.

### 3.5 Characteristics of innate immune cells between the two groups

**Figure 5:**
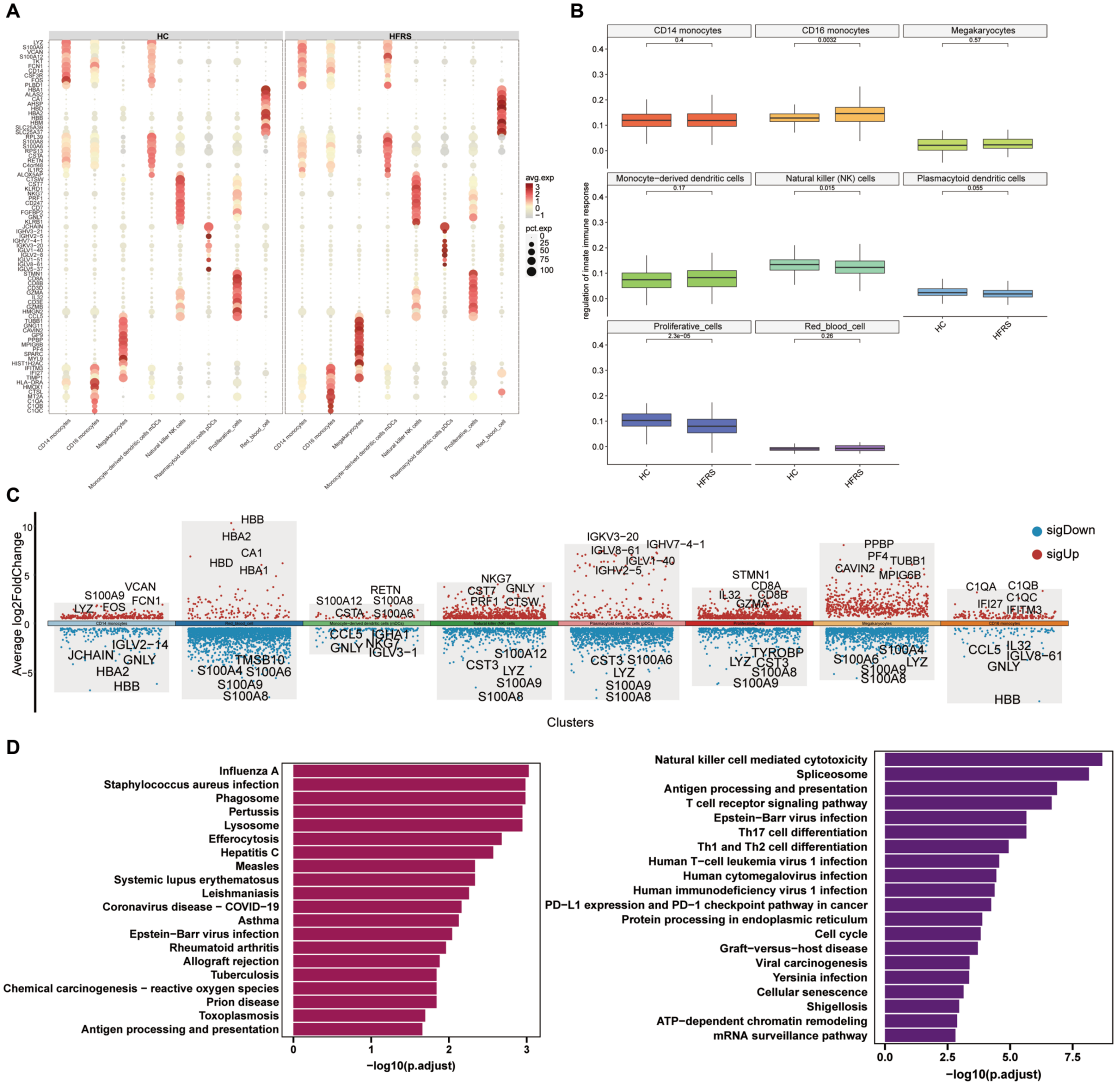
Characteristics of Innate Immune Cells in the HC and HFRS Groups. **(A)** The bubble plot illustrates the expression levels and expression percentages of the top 70 differentially expressed genes (DEGs) in each cell subtype. The size of each bubble corresponds to the percentage of expression in the cell, while the color represents its expression level. **(B)** Box plots are used to visualize the cell state scores of genes associated with innate immune response regulation in various cell subtypes. The box plots are divided into two different groups: HC and HFRS. They describe the comparison of cell state scores between the two groups. Each condition is represented by a different color, and the y-axis represents the median. **(C)** The plot illustrates the differentially expressed genes (DEGs) between the two groups in various cell subtypes, and marks the top five upregulated or downregulated genes based on the absolute value of Log2Foldchange. **(D)** KEGG enrichment analysis of the DEGs in CD16 monocytes and NK cells was conducted using the KEGG database.

To delve deeper into the transcriptional alterations within innate immune cells between the HC and HFRS cohorts, we scrutinized the expression profiles of innate immune-related cell subtypes. Leveraging the UMAP findings, we delineated two principal categories of innate immune cells based on characteristic gene markers, namely CD14 monocytes and CD16 monocytes (Figure 5A). Through an analysis of differentially expressed genes (DEGs) in each cell subtype across both groups, we unveiled a diverse array of gene ontologies. Notably, a compelling divergence in the expression of 70 genes implicated in innate immune responses emerged between the two cohorts (Figure 5C). Functional enrichment analysis illuminated that the distinct genes associated with CD16 monocytes were intricately linked with pathways encompassing influenza A, Phagosome, Pertussis, and Lysosome. Conversely, the distinct genes correlated with NK cells were implicated in pathways including Natural killer cell-mediated cytotoxicity, Spliceosome, Antigen processing and presentation, TCR signaling pathway, Epstein–Barr virus infection, and Th17 cell differentiation (Figure 5D). Furthermore, our observations revealed a pronounced augmentation in the innate immune response of CD16 monocytes in the HFRS cohort relative to the HC group, juxtaposed against a notable attenuation in the innate immune response of NK cells (Figure 5B). In summation, these findings delineate a compromised innate immune response in HFRS.

### 3.6 Characterization of T cells

**Figure 6:**
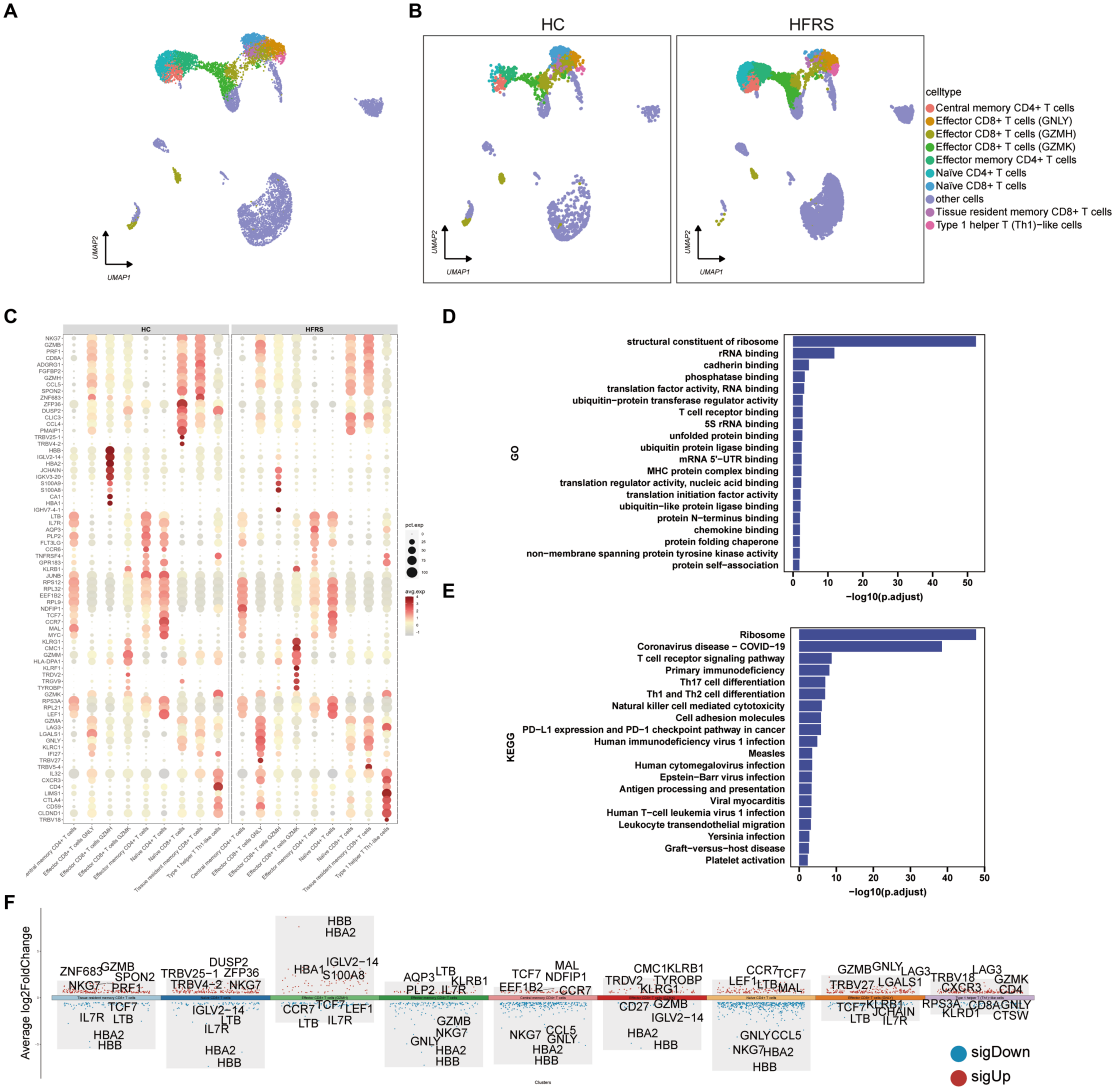
DEGs in T cells in the HC and HFRS groups. **(A)** The UMAP visualization of distinct populations of Central memory CD4 + T cells, Effector CD4+ T cells, Effector CD8+ T cells (GNLY), Effector CD8+ T cells (GZMH), Effector CD8+ T cells (GZMK), Naive CD4+ T cells, Naive CD8+ T cells Effector memory CD4+ cells, Tissue resident memory CD8+ T cells and Type 1 helper T like cells depicted. Additional cell clusters are denoted as “other cells.” Each data point represents an individual cell, and its color signifies the cell type. **(B)** The UMAP visualization of T cell subset acquired from the HC group (*n* = 2) and the HFRS group (*n* = 6) is depicted. Each data point corresponds to a distinct cell, with its color denoting the specific cell type. **(C)** The bubble plot illustrates the expression levels and expression percentages of the top 70 differentially expressed genes (DEGs) in each cell subtype. The size of each bubble corresponds to the percentage of expression in the cell, while the color represents its expression level. **(D)** Enrichment analyses of the DEGs were performed using the BP database within the GO. The GO terms are annotated with their respective names and IDs, and are arranged in descending order based on the logarithm of the reciprocal of the *p* value (−log10). The top 20 enriched GO terms are displayed. **(E)** Enrichment analyses of the KEGG were performed. The top 20 enriched GO terms are displayed. **(F)** The plot illustrates the differentially expressed genes (DEGs) between the two groups in various cell subtypes, and marks the top five upregulated or downregulated genes based on the absolute value of Log2Foldchange.

To elucidate the alterations in T cells following the onset of HFRS, a thorough investigation of T cells within PBMCs was conducted. Leveraging canonical T cell markers, a UMAP visualization revealed a total of nine distinct T cell subsets (Figures 6A,B). Through an analysis of differentially expressed genes (DEGs) in monocytes from both groups, an intricate gene ontology was constructed, highlighting notable differences in the expression of 70 genes related to immune response between the two cohorts (Figure 6C). Functional enrichment analysis based on Gene Ontology (GO) showcased the predominant involvement of T cells in various biological processes, including structural constituents of ribosome, rRNA binding, cadherin binding, translation factor activity, RNA binding, and phosphatase binding (Figure 6D). Moreover, KEGG pathway enrichment analysis revealed the participation of T cells in pathways such as Ribosome, Primary Immunodeficiency, TCR signaling pathway, Th17 cell differentiation, Th1 and Th2 cell differentiation (Figure 6E). The expression profiles of DEGs in T cells are graphically depicted, with red indicating upregulation and blue indicating downregulation, while the top five genes exhibiting the most significant changes are highlighted (Figure 6F).

### 3.7 Characterization of B cells

**Figure 7:**
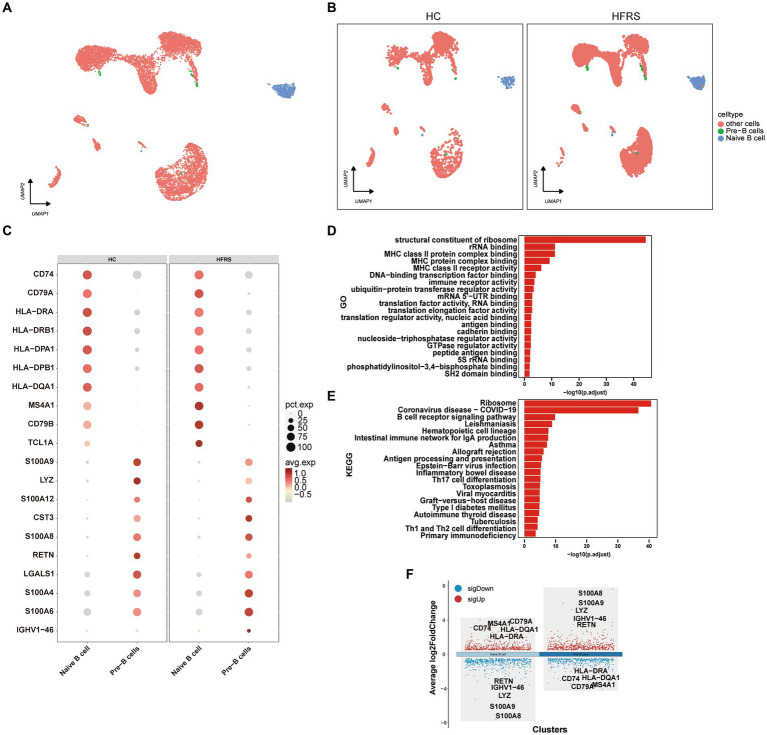
DEGs in B cells in the HC and HFRS groups. **(A)** The UMAP visualization of distinct populations of Naive B lymphocytes and Pre-B lymphocytes is depicted. Additional cell clusters are denoted as “other unidentified cells.” Each data point represents an individual cell, and its color signifies the cell type. **(B)** The UMAP visualization of B cell subset acquired from the HC group (*n* = 2) and the HFRS group (*n* = 6) is depicted. Each data point corresponds to a distinct cell, with its color denoting the specific cell type. **(C)** The bubble plot illustrates the expression levels and expression percentages of the top 70 differentially expressed genes (DEGs) in each cell subtype. The size of each bubble corresponds to the percentage of expression in the cell, while the color represents its expression level. **(D)** Enrichment analyses of the DEGs were performed using the Biological Process (BP) database within the GO. The GO terms are annotated with their respective names and IDs, and are arranged in descending order based on the logarithm of the reciprocal of the *p* value (−log10). The top 20 enriched GO terms are displayed. **(E)** Enrichment analyses of the KEGG were performed. The top 20 enriched GO terms are displayed. **(F)** The plot illustrates the differentially expressed genes (DEGs) between the two groups in various cell subtypes, and marks the top five upregulated or downregulated genes based on the absolute value of Log2Foldchange.

To characterize the modifications in B lymphocytes subsequent to the initiation of HERS, a comprehensive examination of B lymphocytes within PBMCs was conducted. By employing the expression of canonical B lymphocyte markers, a UMAP visualization generated a total of two distinct B lymphocyte subpopulations (Figures 7A,B). Through an exploration of DEGs in monocytes from both cohorts, an extensive gene ontology was established, highlighting noteworthy disparities in the expression of 70 genes related to immune response between the two groups (Figure 7C). The outcomes of the functional enrichment analysis, utilizing GO, elucidated the prominent role played by B lymphocytes in diverse biological processes including the structural constituent of ribosome, rRNA binding, MHC class II protein complex binding, MHC protein complex binding, and ribonucleoprotein complex binding (Figure 7D). Additionally, the KEGG pathway enrichment analysis indicated the involvement of B lymphocytes in various pathways such as the Ribosome, Epstein–Barr virus infection, Spliceosome, and Intestinal immune network for IgA production (Figure 7E). The expression profile of DEGs in T lymphocytes is depicted, with red indicating upregulation and blue indicating downregulation. The figure showcases the top five genes demonstrating the most substantial alterations (Figure 7F).

### 3.8 hdWGCNA identifes the hub genes of T cell related to HFRS

**Figure 8:**
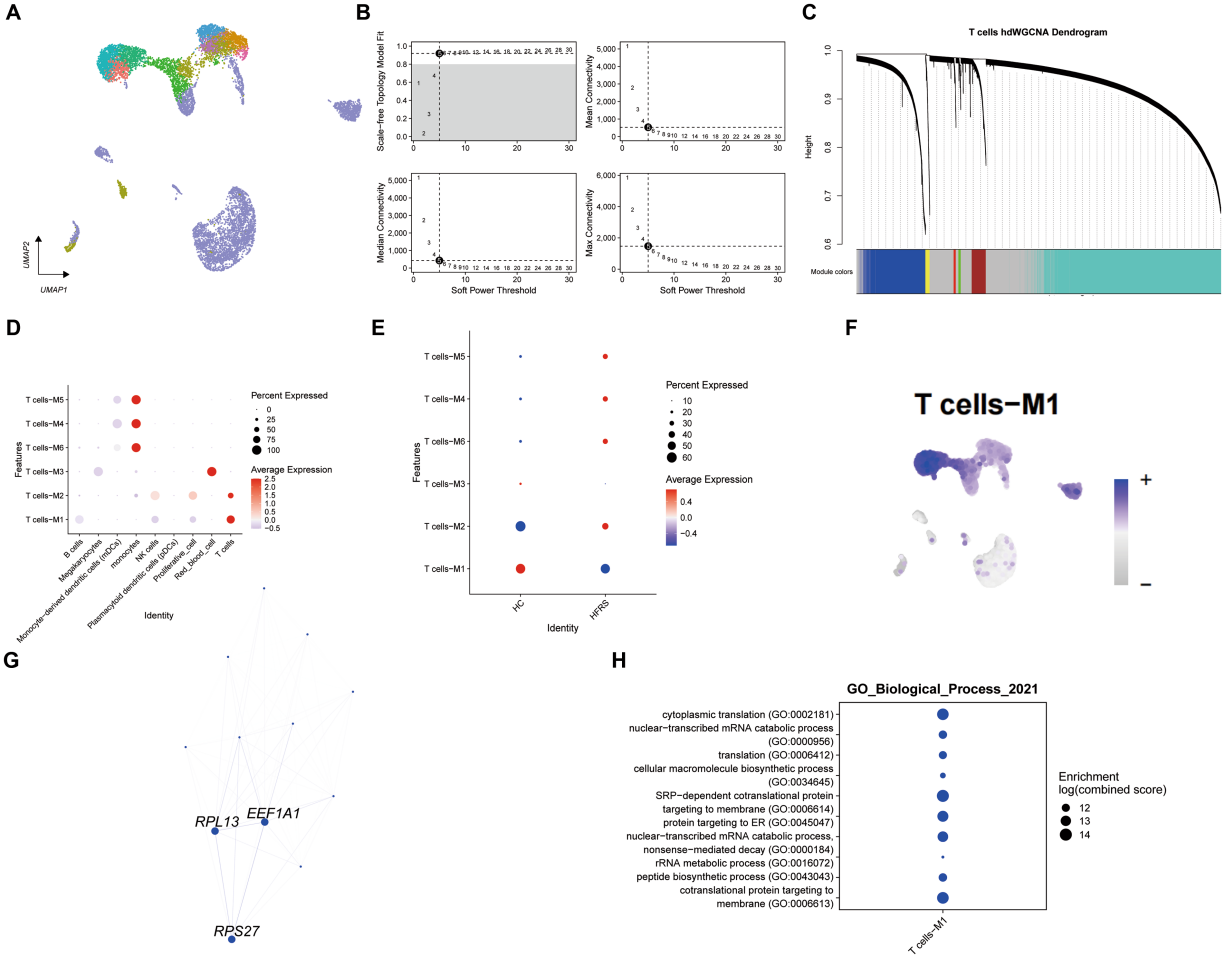
Identification of gene co-expression modules among T cells. **(A)** Unsupervised clustering and annotation of cell types in Healthy and HFRS samples. **(B)** Power value equal to 5 when the network reached a scale-free distribution. **(C)** Highly variable genes were clustered into 6 modules through hdWGCNA. **(D)** Dot plot for enrichment of modules in different cell types. **(E)** Dot plot for enrichment of modules in different groups. F. UMAP of the expression of T cells -M1 among all cells. **(G)** The protein–protein interaction network illustrates the interactions within the T cell module M1, with the top three hub genes showing the most interactions highlighted in the graph. **(H)** Dot plot of the GO functional enrich analysis of the module T cells -M1.

Employing high-dimensional weighted gene co-expression network analysis, we unveiled the primary molecular characteristics of T cells. Utilizing a soft threshold of five, we constructed an unscaled network to optimize connectivity, resulting in the discernment of six gene modules (depicted in Figures 8A–C). Remarkably, modules T cells -M1 and T cells -M2 exhibited significant enrichment in T cells. Upon juxtaposition of the HFRS and healthy control cohorts, a conspicuous reduction in the enrichment of the T cells – M1 module within the HFRS group was noted (as illustrated in Figures 8D,E). Ucell scores were calculated across all cells for each of the seven modules, with modules T cells -M1 displaying the highest scores among T cells (as portrayed in Figure 8F). Subsequently, we constructed a protein–protein interaction (PPI) network, revealing interactions among pivotal genes within the T cells -M1 modules (depicted in Figure 8G). These pivotal genes participate in processes pertinent to T helper 1 (Th1) cell-specific transcription factors, protein synthesis, and more, encompassing EEF1A1, RPL13, and RPS27, and they occupy central positions within the network. Furthermore, GO enrichment analysis on genes within the T cells – M1 module revealed their involvement in pathways associated with SRP − dependent cotranslational protein targeting to membrane (as shown in Figure 8H). In summary, our investigation delved into gene expression modules within T cells and identified pivotal hub genes implicated in the pathogenesis of HFRS.

### 3.9 hdWGCNA identifes the hub genes of B cell related to HFRS

**Figure 9:**
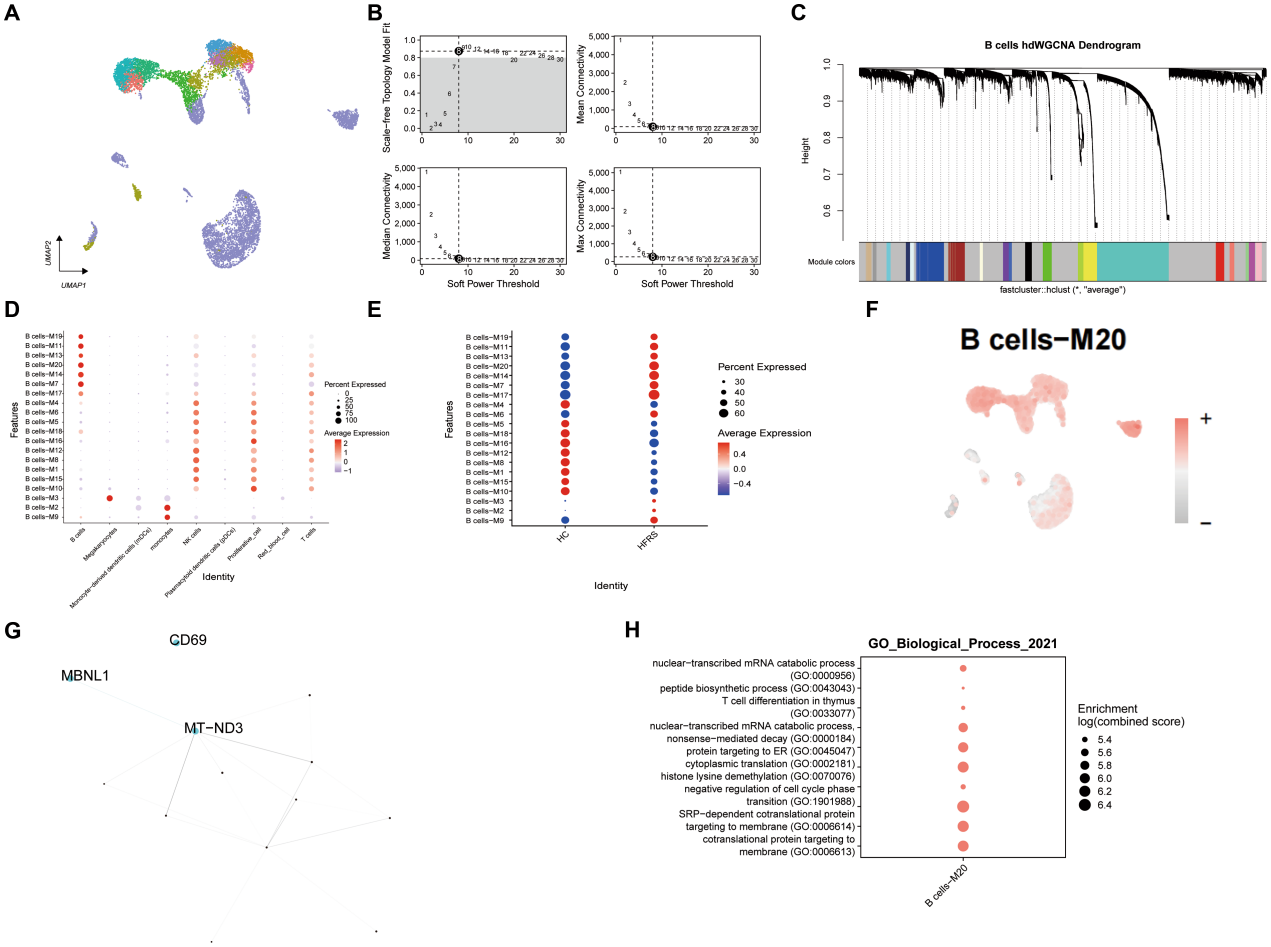
Identifcation of gene co-expression modules among B cells. **(A)** Unsupervised clustering and annotation of cell types in Healthy and Sepsis samples. **(B)** Power value equal to 8 when the network reached a scale-free distribution. **(C)** Highly variable genes were clustered into 20 modules through hdWGCNA. **(D)** Dot plot for enrichment of modules in different cell types. **(E)** Dot plot for enrichment of modules in different groups. **(F)** UMAP of the expression of B cells -M20 among all cells. **(G)** The protein–protein interaction network illustrates the interactions within the B cell module M20, with the top three hub genes showing the most interactions highlighted in the graph. **(H)** Dot plot of the GO functional enrich analysis of the module B cells -M20.

Utilizing hdWGCNA, we elucidated the principal molecular features characterizing B cells. Employing a soft threshold of eight, we constructed an unscaled network tailored for B cells to optimize connectivity, effectively identifying twenty modules (illustrated in Figures 9A–C). Particularly noteworthy, modules B cells -M20 exhibited notable enrichment within B cells. Upon comparison with the healthy control cohort, a significant reduction in the enrichment of the B cells – M20 module was evident in the HFRS group (as illustrated in Figures 9D,E). Assessing the Ucell scores across all cells for these seven modules, we observed module B cells -M20 achieving the highest score within B cells (depicted in Figure 9F). Subsequently, we established a Protein–Protein Interaction (PPI) network, revealing the interplay among pivotal genes within module B cells -M20 (depicted in Figure 9G). These pivotal genes participate in processes concerning respiratory chain NADH dehydrogenase synthesis and mediation of pre-mRNA alternative splicing regulation, exemplified by MT − ND3 and MBNL1, occupying central positions within the network. Furthermore, GO enrichment analysis conducted on genes within the B cells – M20 module unveiled their involvement in pathways associated with negative regulation of cell cycle phase, cotranslational protein targeting to membrane, and nuclear−transcribed mRNA catabolic process (Figure 9H). In conclusion, our investigation adeptly explored gene expression modules within B cells, ultimately pinpointing pivotal hub genes contributing to the pathogenesis of HFRS.

### 3.10 Cell-cell interaction analysis related to T cells in scRNA-seq

**Figure 10:**
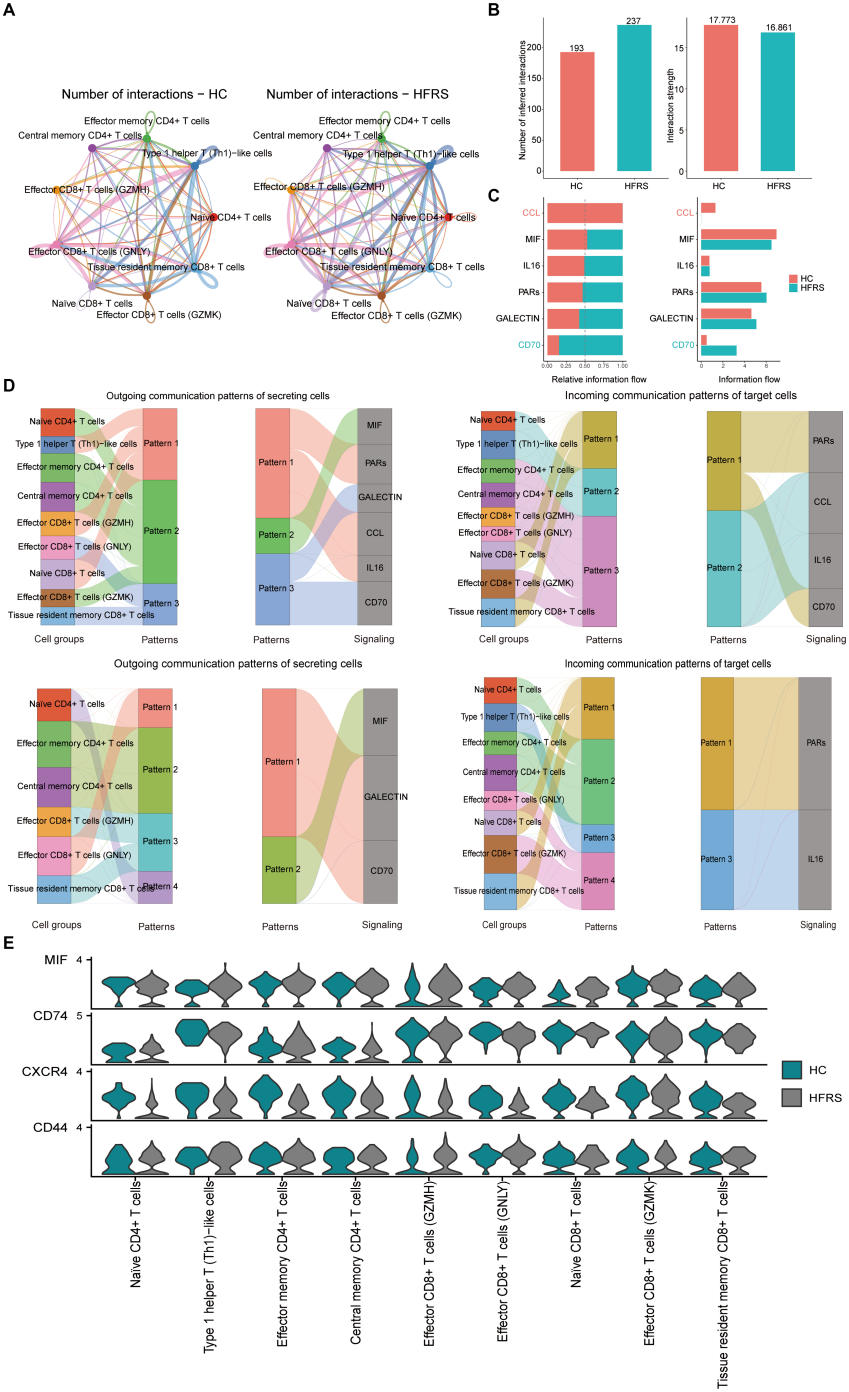
CellChat analysis of the communications between T cell subsets. **(A)** Circle plots illustrating the number and strength of interactions between T cell subsets in HC and HFRS group. **(B,C)** Identification of singling roles for cells using network centrality analysis. **(D)** The Sankey diagram of signaling pathways in different patterns for various cell types in the HC and HFRS groups. The left panel illustrates how cells coordinate with each other as signal senders and how they coordinate with certain signaling pathways to drive communication under outgoing patterns. The right panel illustrates how cells coordinate with each other as signal receivers and how they coordinate with certain signaling pathways to respond to incoming signals under incoming-patterns. The thickness of the flow indicates the contribution of the cell group or signaling pathway to each latent pattern. **(E)** Violin plot showing the expression distribution of signaling genes involved in the inferred signaling network between normal and HFRS group.

In our investigation, we employed the CellChat (version 1.6.1) R package to delve into cell–cell communication intricacies. This tool facilitated the exploration of ligand-receptor interactions on the cell surface, offering insights into intercellular information transmission (12). Leveraging gene expression data, we deduced protein expression and established a comprehensive cell interaction network. Extracting the expression matrix and cell classification information, we utilized the “createCellChat” function to generate a cell chart object, from which we computed the communication probability to infer the cell interaction network. To ensure data fidelity, we filtered out communication relationships involving low-quality cells, setting a threshold of a minimum of three cells. Additionally, we scrutinized cell communication at the signal pathway level, enabling us to deduce communication pathways between cells. By aggregating cells, we computed the communication network for this higher-level analysis. The communication results showed that in the HC group, the number and intensity of communication were 193 and 17.773, respectively, while in the HFRS group, they were 237 and 16.861, respectively (Figures 10A,B). Furthermore, we observed significant variations in the CD70, MIF, and GALECTIN signaling pathways between the two groups, emphasizing the critical role of CD70, MIF, and GALECTIN signaling pathways in HFRS (Figure 10C). Next, through pattern recognition using CellChat, we predicted coordinated responses between cells. In the HC group, naive CD8+ T cells and Tissue resident memory CD8+ T cells belong to pattern 3, coordinating outward signaling pathways of CD70 and GALECTIN, while naive CD8+ T cells and Tissue resident memory CD8+ T cells belong to pattern 1, coordinating receiving pathways of CD70 and PARs signaling. Conversely, in the HFRS group, effector CD8+ T cells (GNLY) belong to pattern 1, coordinating outward signaling pathways of CD70 and GALECTIN, while naive CD8+ T cells and Tissue resident memory CD8+ T cells belong to pattern 1, coordinating receiving pathways of PARs signaling (Figure 10D). Hence, it can be inferred that the communication patterns among cells undergo alteration in the diseased condition. It is noteworthy that the expression signals of MIF signaling pathway in each cell group were not significantly different (Figure 10E). These results reveal the coordination of functions among multiple cell groups and signaling pathways, as well as variations in the number and intensity of signals.

### 3.11 Pseudo-time analysis and transcription factor prediction of T cells

**Figure 11:**
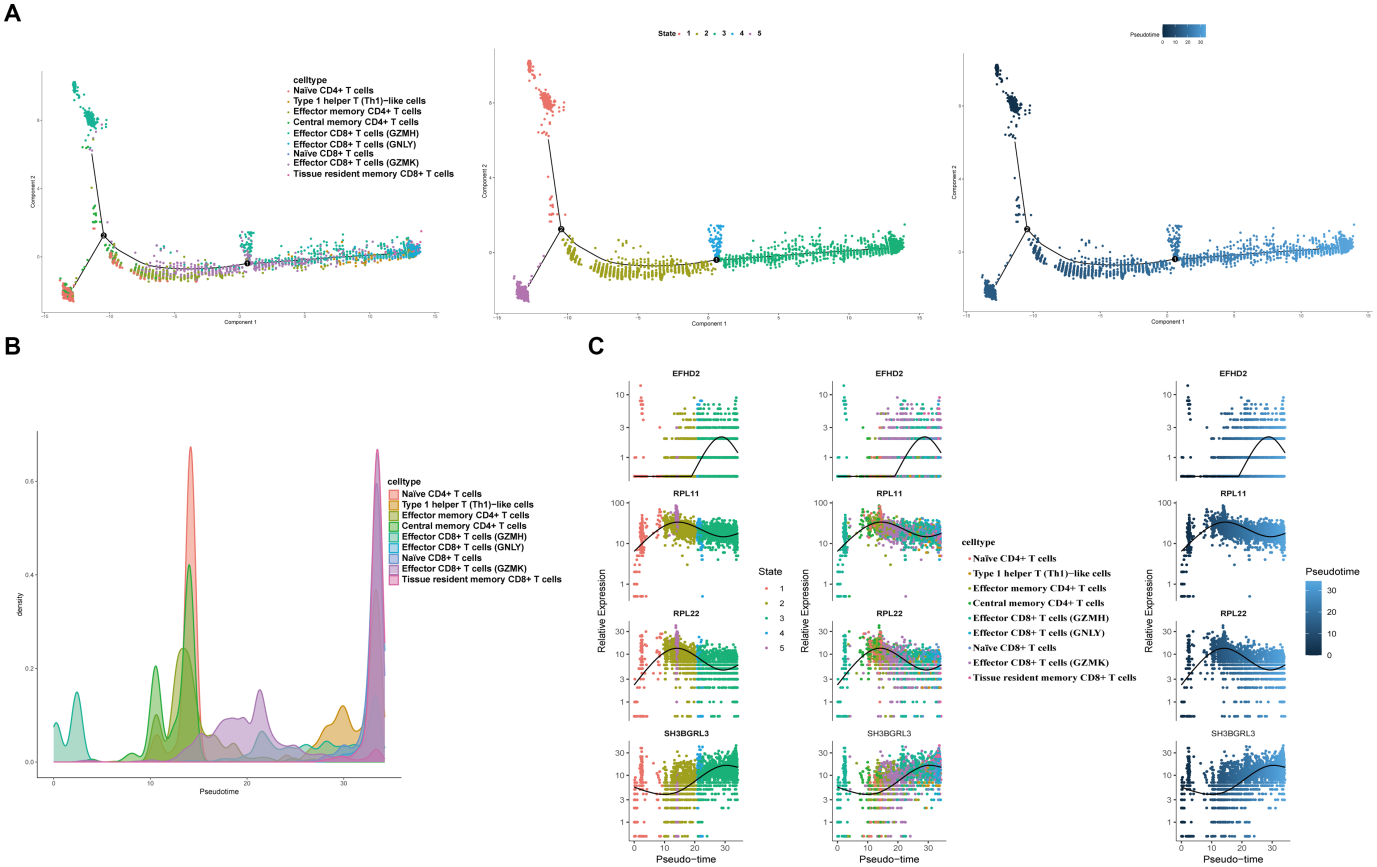
Characterization of the landscape of T cells and developmental trajectories of T cells in HFRS. **(A)** The pseudo-time distribution of different T cell subtypes, the left panel depicts each cell subtype marked with a different color along the pseudo-temporal trajectory, the middle panel illustrates five temporal states of annotated cell development, the right panel represents the trend trajectory of development, where deeper shades of blue indicate earlier developmental time points. **(B)** Cell density variation of T cell subtypes during the pseudotime. **(C)** Pseudo-scatter plots showing the expression variation and distribution of some specific genes during the pseudotime, color-coded by cell types.

**Figure 12:**
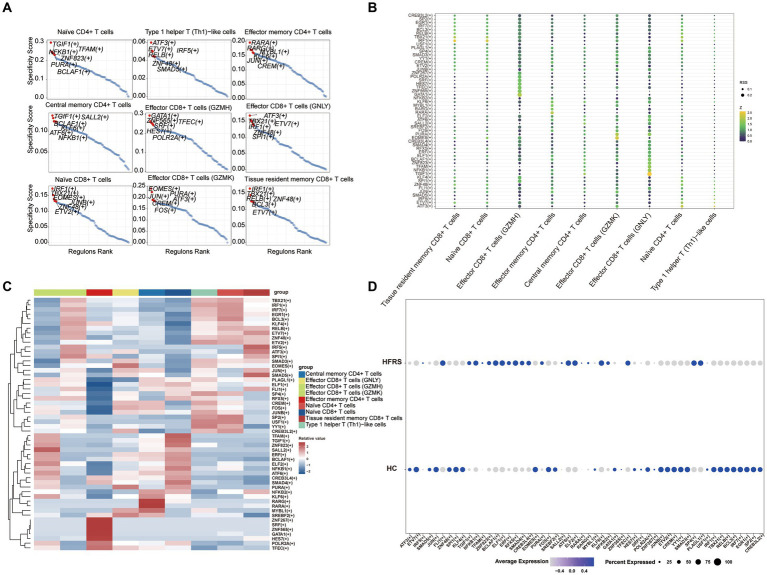
The T cells of TF predicted by SCENIC analysis. **(A)** RANK plot of T cell subgroups TFs. **(B)** Dimplot of T cell subgroups main TFs. **(C)** Heatmap of expression levels of selected TFs in T cell subgroups. **(D)** Dimplot of T cell of TFs in the differential analysis between HFRS and HC.

To elucidate the developmental stages of T cell subsets with greater certainty, we employed the Monocle2 package to conduct pseudotime series analysis. The trajectory analysis unveiled that the majority of cell subsets progress through a singular developmental stage. Notably, within these clusters, Effector CD8+ T cells (GZMH) were positioned at the initial phase of the trajectory, subsequently differentiating into Central memory CD4+ T cells, Effector CD4+ T cells, Effector CD8+ T cells (GNLY), Effector CD8+ T cells (GZMK), Naïve CD4+ T cells, Type 1 helper T (Th1)-like cells, and Tissue resident memory CD8+ T cells (Figures 11A,B). Furthermore, we observed a progressive increase in the expression levels of EFHD2, RPL11, and SH3BGRL3 during the course of cell differentiation (Figure 11C). Subsequently, we employed SCENIC analysis to predict transcription factors (TFs) in T cells, visualizing the results using R. The findings indicated elevated expression levels of transcription factors in Effector CD8+ T cells (GZMH), Effector CD8+ T cells (GZMK), and Naïve CD4+ T cells (Figures 12A–C). Notably, transcription factors NFKB and JUN, implicated in the regulation of immune inflammation, exhibited heightened expression in Naive CD4+ T cells from the HFRS group compared to healthy controls. These observations shed light on the mechanisms underlying the inflammatory activation of Naive CD4+ T cells in the context of the disease (Figure 12D).

### 3.12 Pseudo-time analysis and transcription factor prediction of B cells

**Figure 13:**
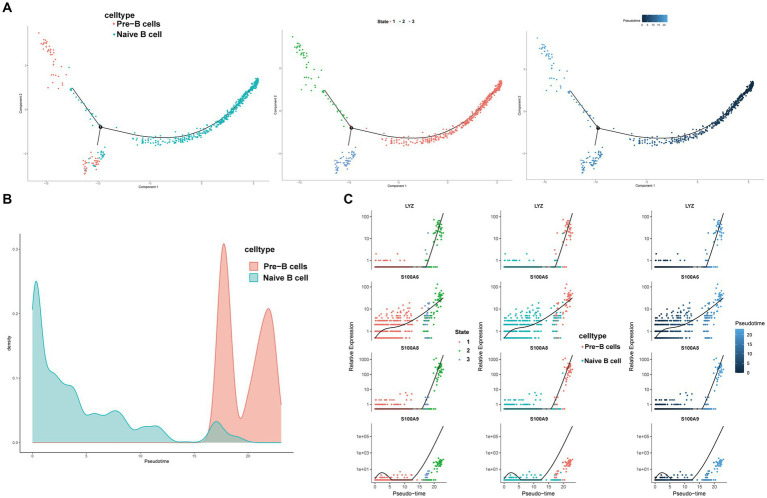
Characterization of the landscape of B cells and developmental trajectories of B cells in HFRS. **(A)** The pseudo-time distribution of different B cell subtypes, the left panel depicts each cell subtype marked with a different color along the pseudo-temporal trajectory, the middle panel illustrates five temporal states of annotated cell development, the right panel represents the trend trajectory of development, where deeper shades of blue indicate earlier developmental time points. **(B)** Cell density variation of B cell subtypes during the pseudotime (top). **(C)** Pseudo-scatter plots showing the expression variation and distribution of some specific genes during the pseudotime, color-coded by cell types.

**Figure 14:**
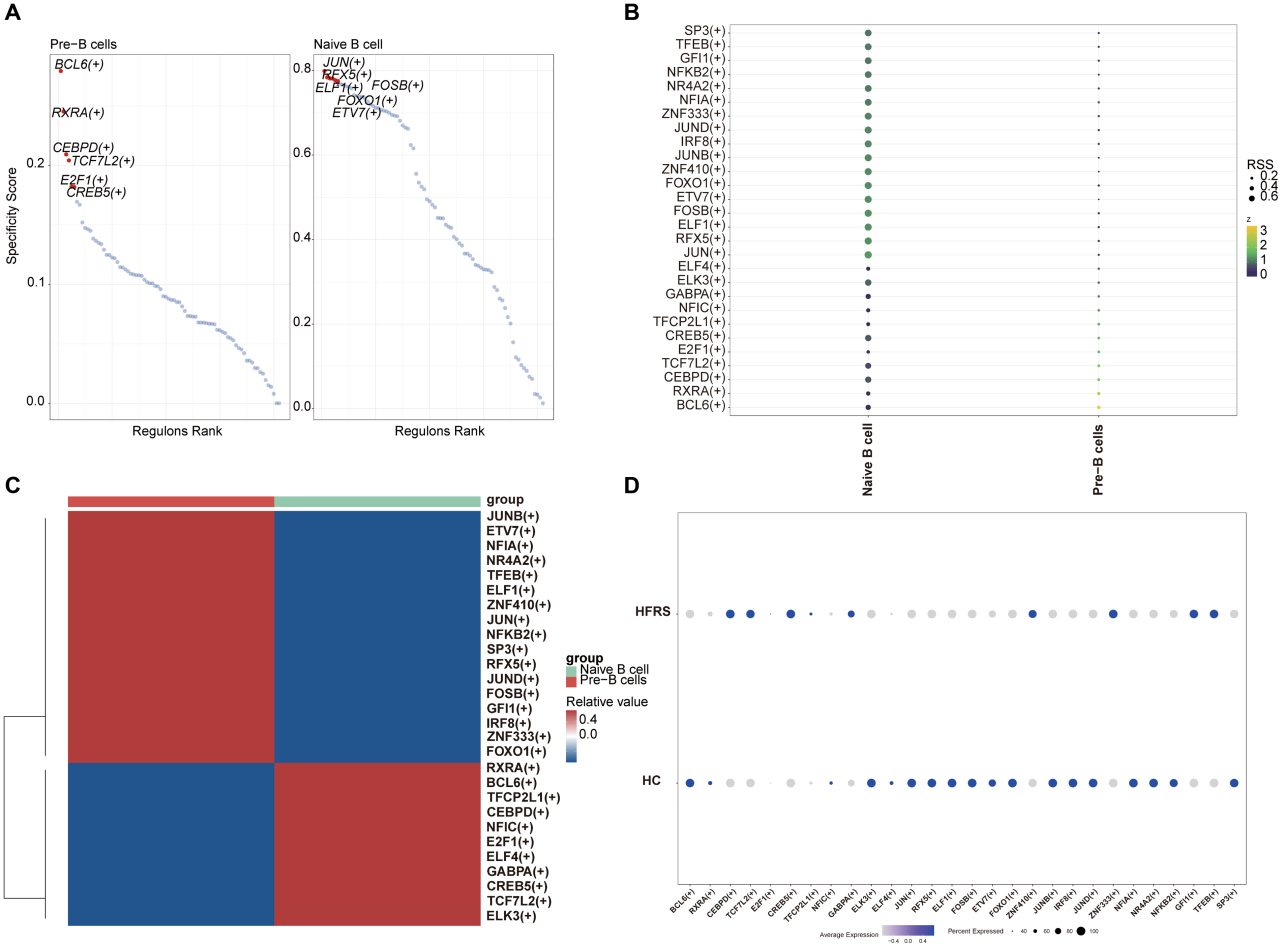
The B cells of TF predicted by SCENIC analysis. **(A)** RANK plot of B cell subgroups TFs. **(B)** Dimplot of B cell subgroups main TFs. **(C)** Heatmap of expression levels of selected TFs in B cell subgroups. **(D)** Dotplot of B cell of TFs in the differential analysis between HFRS and HC.

We conducted pseudotime trajectory analysis of B cell clusters using Monocle2 to delineate the developmental trajectory of B cells, as well as to characterize the branching distribution and cell density of each cluster. Within these clusters, naïve B cells were situated at the initial segment of the trajectory, subsequently differentiating into Pre-B cells (Figures 13A,B). As the B cell subsets progressed through differentiation, we observed a progressive rise in the expression levels of genes associated with the LYZ and S100A families (Figure 13C). The SCENIC analysis facilitated the prediction of transcription factors (TFs) in B cells, with visualization conducted using R. The findings indicated a notable expression of transcription factors in Naïve B cells (Figures 14A–C). Particularly, immune-inflammation regulators NFKB and JUN exhibited heightened expression primarily in Naive B cells within the HFRS group compared to the healthy group, elucidating the mechanism of inflammatory activation of B subset cells in the context of the disease (Figure 14D).

## 4 Discussion

In this investigation, we have uncovered significant insights into the gene expression disparities associated with HFRS. Initially, leveraging extensive RNA sequencing data and TRUST4 analysis, we constructed comprehensive TCR and BCR repertoires. The pronounced variability observed in the CDR3 and the divergences in amino acid sequences stem from the recombination of Variable (V), Diversity (D), and Joining (J) genes in T-cell and B-cell receptors (13). This V(D)J gene recombination stands as a hallmark of adaptive immunity, empowering the immune system to mount effective responses against a myriad of antigens. Prior investigations have postulated that the attributes of TCR and BCR repertoires might furnish insights into the etiology of autoimmune disorders (14, 15).

Our findings have unearthed notable disparities in the diversity of CDR3 amino acid sequences between the HFRS cohort and the HC cohort. This further supports our previous hypothesis positing that autoimmune maladies may precipitate an upsurge in specific CDR3 amino acid sequences. Moreover, our analysis has pinpointed substantial alterations in the TRBV, TRBJ, IGHV, and IGHJ genes, a revelation scarcely documented in previous studies. These findings help fill gaps in our understanding of the attributes of TCR and BCR repertoires, suggesting that HFRS patients might mount immune responses against commonplace autoantigens. Such discernments hold profound implications for the refinement of targeted biologics and the diagnostic strategies employed in managing HFRS.

We integrated multiple scRNA-seq datasets using currently popular single-cell sequencing techniques (16, 17). Comparative analysis revealed notable alterations in the composition of immune cell subsets between the HFRS and HC groups. Specifically, the HFRS group exhibited a significant decrease in the proportion of CD8+ T cells compared to the HC group, while the proportion of CD16 monocytes was notably elevated in the HFRS group. These findings are consistent with previous studies (18, 19). It is speculated that targeting CD8+ T cells and CD16+ monocytes could potentially alleviate the pathogenesis of HFRS. We examined genes differentially expressed in T cells and B cells between the HC and HFRS groups and found that their functional and enrichment analyses illustrate immune-related pathways. The signaling pathways involved in T cells and B cells, including Epstein–Barr virus infection, Spliceosome, Intestinal immune network for IgA production, Primary immunodeficiency, TCR signaling pathway, Th17 cell differentiation, and Th1 and Th2 cell differentiation, may indicate a synergistic effect between all immune cells in the peripheral blood of HFRS patients.

To elucidate the related genes involved in disease regulation in T and B cells further, we analyzed the characteristics of gene co-expression regulatory modules during the development of HFRS using the hdWGCNA method. The results revealed a significant decrease in the enrichment of the T cell-M1 module in HFRS patients, primarily involving the regulation of ribosome biogenesis and immune cell activation. Additionally, the B cell-M2 module was predominantly associated with cell proliferation and RNA transcriptional regulation, highlighting the crucial roles of T cells and B cells in the pathogenesis of HFRS (20, 21). Furthermore, immune cell activation heavily relies on the cell’s intrinsic transcriptional regulatory state. Therefore, changes in cellular transcriptional levels may be both a cause and a consequence of alterations in immune function. Consistent with our findings, our study suggests that the core genes in the T cell-M1 module are involved in Th1 cell transcriptional activation. Therefore, transcriptional regulation that promotes immune activation may be a key mechanism leading to immune dysfunction in HFRS patients.

Next, we used the “CellChat” software package to investigate the specific signaling roles played by each cell group, the intricate cellular communication network within complex tissues, and to explore the relevance to HFRS by calculating communication probabilities (22). Our results reveal a key role in MIF signaling in HFRS. Macrophage migration inhibitory factor, a proinflammatory cytokine encoded within a functional polymorphic genetic locus, has been identified as a multipotent key cytokine secreted by many other cell types involved in immune responses and physiological processes (23, 24). Studies have shown that high expression levels of MIF are associated with the severity of clinical phenotypes in a variety of autoimmune and inflammatory diseases such as rheumatoid arthritis, asthma, and systemic sclerosis (25). MIF interacts with its receptors CD74/CD44, CXCR2, CXCR4, and CXCR7 in an autocrine and paracrine manner (26). The important thing to note is that there was no significant change in MIF in our results, which could be attributed to the small sample size resulting in the lack of noticeable differences. In addition, recent *in vivo* studies have revealed multiple unique properties of CD70, a member of the tumor necrosis factor receptor superfamily that interacts with ligand CD27. First, CD70 is only transiently expressed on activated T and B lymphocytes, mature killer cells, and mature dendritic cells, with limited expression on normal non-immune cells. Second, the interaction between CD70 and CD27 acts as a costimulatory signal in T and B lymphocyte activation and induces lymphocyte proliferation (27, 28). Therefore, activation of the CD70-CD27 interaction may play a pro-proliferative activity in viral infection. Another player of interest in the complex network of intercellular communication is GALECTIN, a β-galactosidase binding protein that is present in both the nucleus and cytoplasm. GALECTIN is involved in the control of phagocytosis and macropinocytosis (29, 30). In summary, our study suggested that CD70 and GALECTIN may be potential biomarkers or targets for the diagnosis and treatment of HFRS.

In order to further elucidate the developmental stages of T/B cell subpopulations, we conducted pseudotime series analysis using Monocle2 software. The trajectory analysis results show that T cells develop along a major branch, originating from effector CD8+ T cells (GZMH), then differentiating into central memory CD4+ T cells, effector CD4+ T cells, effector CD8+ T cells (GNLY), effector CD8+ T cells (GZMK), naive CD4+ T cells, type 1 helper T (Th1)-like cells, and tissue-resident memory CD8+ T cells. Additionally, we observed that with cell differentiation, the expression levels of EFHD gradually increase. EFHD2 is a crucial regulatory factor for T cell cytotoxicity, activating T cell-mediated pro-inflammatory effects (31). On the other hand, B cells exhibit a bifurcating trajectory, with naive B cells positioned at the starting point of one branch, then differentiating into pre-B cells. In the differentiated state of B cell subpopulations, we found that the expression levels of the S100A gene family gradually increase. S100A proteins belong to a group of low molecular weight proteins that play a crucial role in the regulation of inflammation-related processes in many diseases (32). Moreover, previous studies have indicated a correlation between the expression of the S100A gene, increased immunopositive cells, and stimulation of the nuclear factor NFKB signaling pathway (33). In transcription factor analysis, we observed a significant activation of the NFKB transcription factor, which participates in the regulation of immune inflammation, in the diseased state. Therefore, Naive CD4+ T cells and Naive B cells are crucial target cells mediating inflammation activation in the progression of the disease, a process that relies on the involvement of the NFKB transcription factor.

In conclusion, the study delineates the immunological characteristics of the TCR and BCR repertoires in HFRS disease. We detected abnormal changes in the composition of immune cells and the transcriptional profiles of individual clusters in HFRS, highlighting the immune infiltrative influence of the peripheral immune environment in the diseased state. Furthermore, we predicted the roles of cell communication signals at the single-cell level in the pathogenesis of HFRS, as well as identified the core genes responsible for disease development. These findings contribute to our understanding of the molecular and cellular basis of peripheral immune cells in HFRS. Overall, this study may contribute to the future development of diagnostic methods and biological therapies for HFRS patients. Additionally, our next steps include expanding the sample size of the study and conducting supplementary experiments to further validate our current research findings.

## Data availability statement

The original contributions presented in the study are included in the article/Supplementary material, further inquiries can be directed to the corresponding author.

## Author contributions

RX: Writing – original draft. MLin: Visualization, Writing – review & editing. MLiu: Data curation, Methodology, Writing – original draft. QM: Funding acquisition, Writing – review & editing.
