# Supplementary figures and images for: Single cells and TRUST4 reveal immunological features of the HFRS transcriptome
Source: Front Med (Lausanne). 2024 May 13;11:1403335. doi: 10.3389/fmed.2024.1403335 (PMC11128564; doi:10.3389/fmed.2024.1403335)

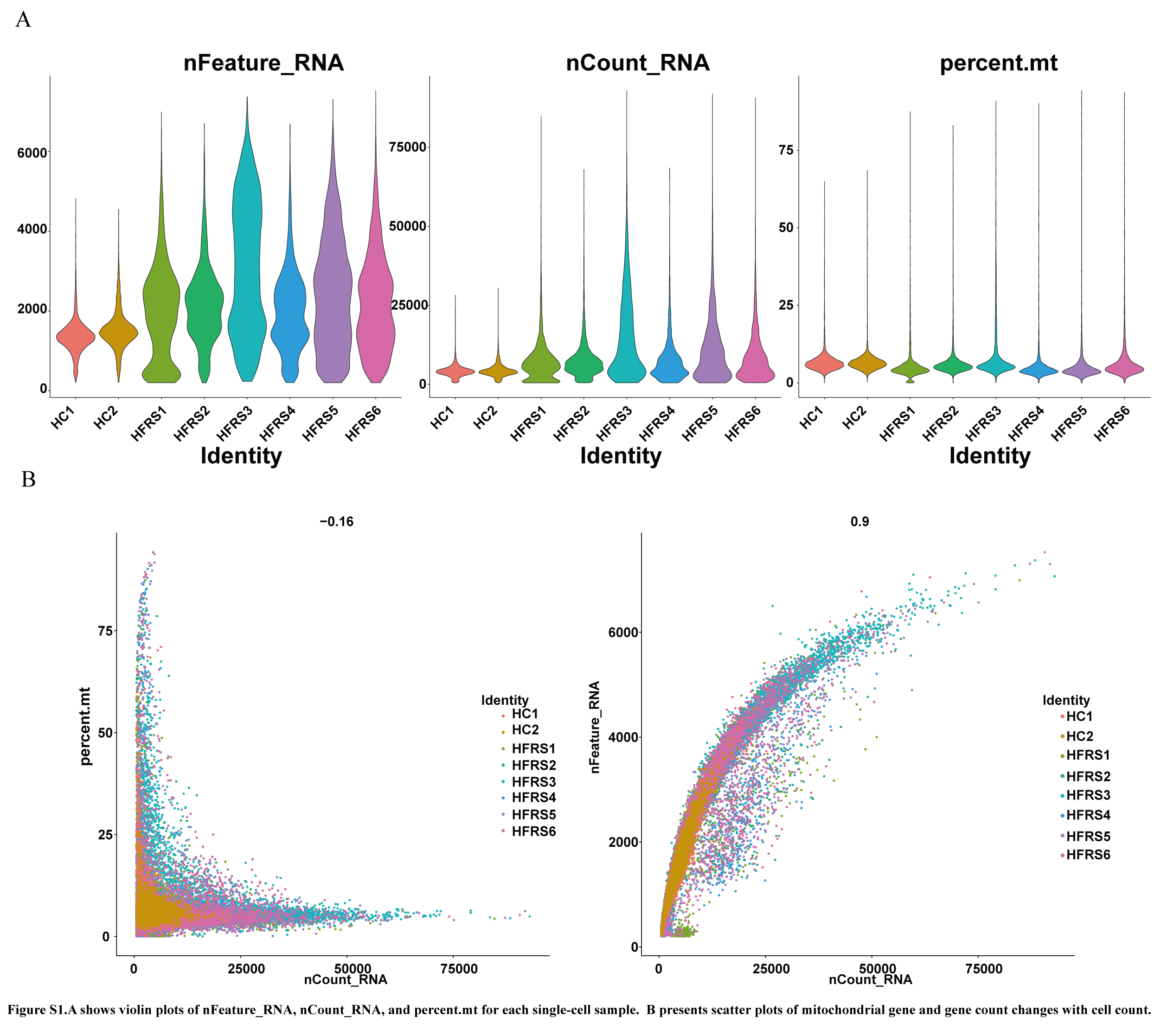

Supplement: Supplementary file 1 [file Image_1.TIF]
